# Lowering the Toxicity of Cd to *Theobroma cacao* Using Soil Amendments Based on Commercial Charcoal and Lime

**DOI:** 10.3390/toxics10010015

**Published:** 2022-01-04

**Authors:** Carla Calixta Calva Jiménez, Liliana Valentina Pinedo Fernández, Cristiano E. Rodrigues Reis

**Affiliations:** EARTH University, Las Mercedes, Guacimo 4442-1000, Limon, Costa Rica; ccalva@earth.ac.cr (C.C.C.J.); lpinedo@earth.ac.cr (L.V.P.F.)

**Keywords:** adsorption, cadmium, charcoal, liming, heavy metal

## Abstract

Carbonaceous and calcareous materials are commonly used as amendments to decrease the Cd mobility in contaminated soils. This study evaluated the effect of amendments applied to cocoa seedlings in the greenhouse, considering the mobilization of soil cadmium toward the seedlings as the main response. The experimental conditions considered soil artificially contaminated with Cd at a concentration of 50 mg Cd kg^−1^ and applications of amendments in different treatments with the presence of charcoal dust and calcium carbonate. The charcoal was characterized by microscopy and by adsorption tests, and it proved to be a material with macropores, with a maximum capacity of 8.06 mg Cd g^−1^ and favorable kinetic behavior according to the adjustment of the data obtained to the pseudo-second-order model. The results also showed that the application of liming decreased the mobility of Cd toward the seedlings, with the liming combined with charcoal leading to the absence of Cd in the cocoa seedlings, considering a residual concentration of Cd in the soil of 35 mg Cd kg^−1^. The results, although limited to a small scale, demonstrated the possibility of applying low-cost and easy-to-handle amendments for the control of Cd in cocoa plantations.

## 1. Introduction

Cacao (*Theobroma cacao*) is an important crop for many developing countries within the tropical zone, with countries in the tropics and subtropics of Africa, Latin America, and Southeast Asia as the largest producers in the world [1,2]. Cacao plantations have been considered as an attractive crop due to the relatively low initial capital investment combined with well-established conventional crop protection approaches [2].

Over the past decades, some reports indicated that cacao plants reaching processing centers were contaminated with worrisome levels of heavy metals, including mercury, lead, arsenic, and cadmium [3]. Among these metals, cadmium toxicity has gained particular interest due to the health effects of kidney malfunction and bone deterioration associated with the consumption of contaminated feed and food materials [4]. Cadmium has rapid mobility in cacao plants, and it can be easily absorbed by the roots and accumulate in the seeds, leading to cacao-derived products with toxic concentrations of this heavy metal [5,6]. Cadmium toxicity causes growth-related and metabolic defects, such as browning of plant roots and chlorosis. Such defects lead to modifications in P and N uptake and transport, yielding Fe uptake and decreased nitrogen fixation, affecting the photosynthetic efficiency of the plants [6]. The sources of cadmium in tropical soils are reviewed elsewhere and can be summarized by anthropogenic practices, such as the dumping of mining residues, industrial effluents, coal, and other materials, which, over the years, lead to detectable concentrations in acid soils [4].

Cadmium remediation in tropical soils can be achieved through, among other technologies, the application of liming and adsorbing materials [7,8]. According to Hamid et al. [7], an effective cadmium management practice involves the change of its form, which is dependent on pH, and its immobilization on solid matrices. Such solid materials are usually porous and may facilitate the binding of cadmium ions onto their surface. Some of these materials include activated carbon, biochar, organic wastes, zeolites, and others [7]. These in situ amendments have been described as a viable solution to some of the problems associated with Cd contamination in productive soils. The mechanisms involved in these soil amending processes are based on precipitation, adsorption, ion exchange, and complexation, leading to decreased mobility and bioavailability in soils [9].

Considering that the remediation of Cd-contaminated soils is often laborious and cost-inhibitive, the objective of this work was to evaluate the use of commercial charcoal and lime in lowering the concentration of Cd in cacao grown in tropical soil. Given the relative scarcity of the adsorbing characteristics of vegetable charcoal, which is not as often evaluated as an adsorber for heavy metals in soils, this study also carried out some preliminary characterization in terms of controlled adsorption of Cd ions in aqueous solutions. In this sense, a set of experiments were carried out using Cd-contaminated soils amended with treatments based on charcoal, lime, and the combination of both to evaluate Cd mobility toward different parts of the cacao plant.

## 2. Materials and Methods

### 2.1. Characterization of the Vegetable Charcoal: Microscopic and Adsorption Potential Analyses

Vegetable charcoal was obtained from a local supermarket in the district of Limón in Costa Rica. The charcoal was ground using a manual grinder and sieved through a 10-mesh screen to provide 2 mm or lower charcoal powder and granules. The charcoal was then characterized at the Research Center in Microscopic Structures (CIEMIC) at the University of Costa Rica (San José, Costa Rica) using a Hitachi S-570 Scanning Electron Microscope set to a voltage of 15 kV.

The adsorption potential of the carbon was evaluated using batch adsorption assays with charcoal powder samples and a Cd(NO_3_)_2_·4H_2_O (Sigma–Aldrich, Saint Louis, MO, USA) stock solution at a concentration of 200 mg Cd L^−1^ from which the samples for the respective dilutions of each cluster of experiments were taken from. The adsorption assays were carried out to evaluate its kinetic behavior, the effect of the medium pH on the adsorption process, and the isotherm profile of the adsorption of Cd ions onto the charcoal powder. All the adsorption assays were carried out at 50 mL conical tubes with 40.0 mL of the Cd(NO_3_)_2_·4H_2_O solution and 0.050 g of charcoal. The tubes were placed onto an orbital shaker set at 180 rpm at 25 °C. The supernatant was then recovered using a Whatman #2 paper filter, and the concentration of Cd ions was determined on the liquid phase via complexometric EDTA titration.

The pH assays were conducted using the Cd(NO_3_)_2_·4H_2_O solution at 20 mg Cd L^−1^ under the pH values of 3.5, 5.0, 6.5, and 8.0, which were adjusted using HCl or NaOH solutions at 1.0 mol L^−1^ for 6 h. The kinetic assays were also carried out using a Cd(NO_3_)_2_·4H_2_O solution at 20 mg Cd L^−1^, with sampling times ranging from 0.1 h up to 24 h. The data for the adsorption isotherm analysis were acquired from a set of experiments varying the concentration of the Cd(NO_3_)_2_·4H_2_O solution from 2 up to 200 mg Cd L^−1^ within 6 h of the assay. In all cases, the amount of Cd adsorbed onto the charcoal powder was estimated via a mass balance approach, considering the difference in concentration of Cd ions at the solution used for a given assay and the concentration of Cd ions at the supernatant of such assay, as described in Equation (1):

(1) $$q=\frac{\left(C_{0}-C_{f}\right)V}{m}$$

Equation (1) relates the concentration of Cd adsorbed onto the charcoal powder *q* in mg Cd g^−1^ as being the product of the difference of the initial and final concentration of Cd in mg Cd L^−1^ as *C*_0_ and *C_f_*, respectively, with the volume *V* of the solution used in the assay in L divided by the mass of the charcoal powder *m* used for such assay in g.

### 2.2. Field Evaluation of the Amendments

The field experiments took place at an open greenhouse in Limón province in Costa Rica (10°12′38″ N and 83°35′47″ W, 40 m above sea level). The average temperature of the test site is 25.1 °C, with an average relative humidity of 83.5% and annual precipitation of 3592.2 mm. The soil used in the experiments was characterized as an inceptisol, according to the USDA classification and possessed no detectable total Cd. The soil pH in water was recorded as 5.28.

Three different treatments and a control were evaluated in triplicate in this study. Each experimental condition consisted of a plant pot filled with soil dosed with a solution of Cd(NO_3_)_2_·4H_2_O to provide an initial Cd concentration of 50 mg Cd kg^−1^ soil with cacao seedlings of approximately 40 cm in height. Each plant pot carried approximately 6.75 kg of soil with a moisture content of approximately 19.85%. A total of 12 round plant pots (22 cm height × 22.5 cm diameter, with a superficial area of 397 cm^2^), each corresponding to a repetition of treatment, were placed in the greenhouse, each one being 30 cm apart from each other. The plant pots were assigned random spots on the greenhouse to lower variability bias.

The treatments were based on the presence of charcoal amended to the soil, on the application of superficial lime (agricultural grade CaCO_3_), or both. The estimation of the amount of charcoal is derived from the study of Bian et al. [10], who reported a ratio of 40 t of biochar per ha of Cd-contaminated soil in a field evaluation in China. Following the ratio described by Bian et al. [10], the proportional amount of charcoal powder added to the treatments that required charcoal was 160 g of charcoal powder per plant pot. Charcoal powder was mixed with the soil using perforated cellulose teabags, each one containing approximately 40 g of charcoal powder, which were easy to harvest at the end of the experiment. On the other hand, the estimation for the liming requirement of this soil was based on the development of a liming curve of the soil with CaCO_3_. The estimation of the liming requirement of this soil was 4.2 t of CaCO_3_ per ha of soil, corresponding to 16.7 g of CaCO_3_ per soil plant. CaCO_3_ was applied on the first day of the experiment through a powder dispersion onto the surface of the soil on the plant pot. Each plant pot was irrigated with 150 mL of distilled water every 2 days. The experimental conditions were evaluated for a total of 60 days. pH analysis of the soil was assessed in water [11]. At the end of the experiment, each seedling was cut into the corresponding fractions of roots, stem, and leaves, all of which were thoroughly washed. The soil and the charcoal bags were collected and stored before the subsequent analyses.

Cd ions in soil and charcoal powders were assayed as total Cd based on HNO_3_ digestion of such samples using a microwave-adapted method from Carter and Gregorich [11]. Cd in plant samples was assessed following drying at 60 °C, following microwave digestion, also adapted from Carter and Gregorich [11]. The microwave system used for sample preparation was a Perkin Elmer Titan MPS, with a maximum power of 1500 W. The quantification of Cd in the samples was processed using a Perkin Elmer 8300 Induction Coupled Plasma with Optical Emission Spectroscopy (ICP–OES) system. Statistical analysis was carried out using OriginPro^®^ 16.

## 3. Results

### 3.1. Charcoal Characterization and Cd Adsorption Potential

**Figure 1:**
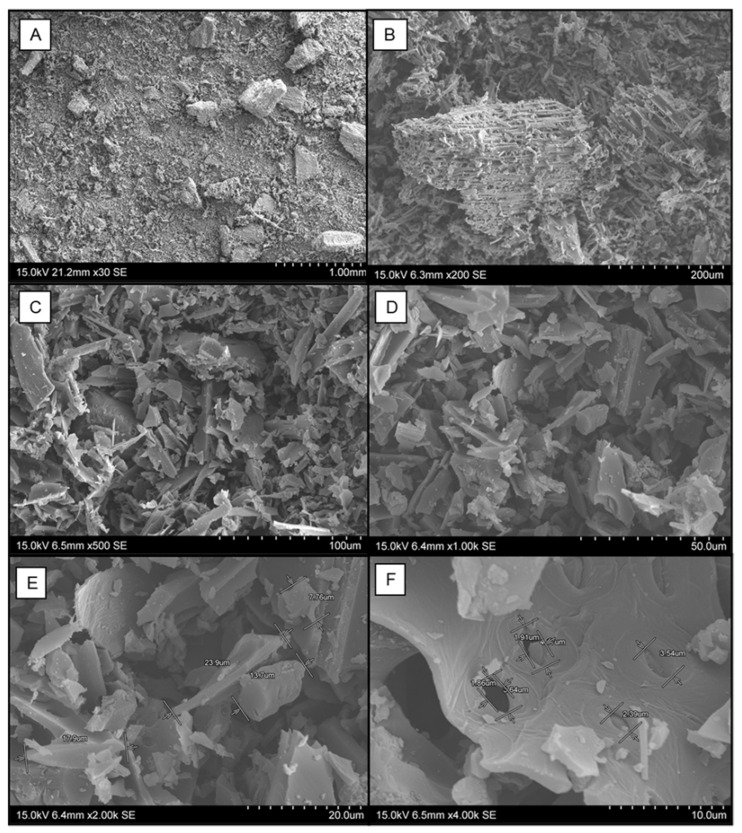
SEM micrograph of the charcoal powder used in the experiments with readings at a 15 kV acceleration voltage under high vacuum and different scales: (**A**) ×30, (**B**) ×200, (**C**) ×500, (**D**) ×1000, (**E**) ×2000, and (**F**) ×4000.

The characterization of the charcoal powder used in the experiments via Scanning Electron Microscopy (SEM) demonstrated the material to be an amorphous and porous material. Figure 1 highlights the micrographs of the charcoal powder under different scales and the dimensioning of typical powder sizes and the diameter of the pores.

As it can be seen from Figure 1C–E, the charcoal powder presented a highly amorphous structure. It can also be seen the disorganized and unstructured morphology, which is typical of carbonaceous materials [12]. Even though the charcoal powder was ground to a 2 mm threshold, from a qualitatively perspective, it can also be seen the wide distribution of the granule size of the powder material. Figure 1E illustrates the sizing of small granules to be as small as 7.76 μm, with other particulates in the micron range, which could lead to a suspension-like fluid behavior in the soil solution. Finally, the diameter of the pores in the larger range granules, observed in Figure 1F, is within a range of 1.8 μm to 4.0 μm. According to the IUPAC classification [13], such pores can be classified as both open and closed pores, within the macro range, since all the recordings are greater than the 50 nm threshold of the definition of macropores. These structures, according to Kurniawan and Ismadji [14], have been demonstrated to be feasible in aiding the adsorption of ions with a kinetic behavior majorly controlled by diffusion mechanisms.

**Figure 2:**
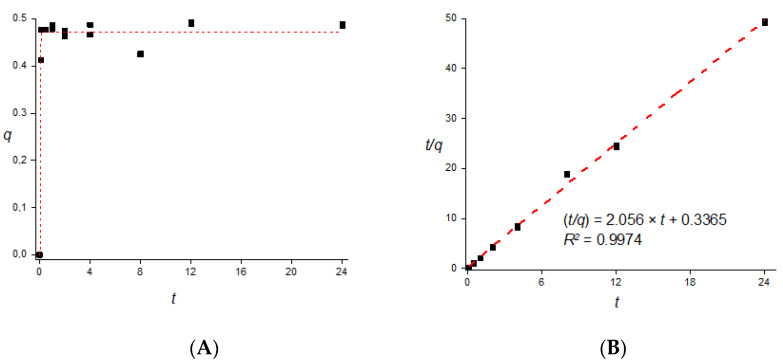
(**A**) Plot of Cd ions adsorbed onto the charcoal powder, measured as q (mg/g) against time (h), illustrating the adsorption kinetic profile of Cd ions onto charcoal powder considering a volume of Cd(NO_3_)_2_·4H_2_O solution of 40.0 mL at a concentration of 20 mg Cd L^−1^ with 0.050 g of charcoal powder. (**B**) Plot of the ratio of the time and the concentration of Cd ions adsorbed onto the charcoal powder, calculated as t/q, in units of h·g/mg against time, measured in h, representing the kinetic data adjusted to the linearized function of pseudo-second-order behavior.

The adsorption behavior of Cd ions was analyzed in terms of the kinetics, the isotherm building, and the medium pH. The kinetic behavior of the adsorption of Cd ions onto the charcoal powder in a controlled environment was demonstrated to be quick and highly efficient under the conditions applied to the study. Figure 2 illustrates the kinetic behavior of the adsorption.

Apart from the fact that there is a plateau concentration range after the first data points, the kinetic plot as described in Figure 2A cannot describe the adsorption behavior of Cd onto charcoal powder from a wider perspective. Therefore, given the considerations from Azizian [15], who described that pseudo-second-order kinetic models often are approachable by adsorbing conditions with low concentrations of the adsorbate, the kinetic data were treated to plot the ratio of time over concentration of Cd on the charcoal powder (*t/q*, with units of h·g_charcoal_·mg^−1^_Cd_) as a function of the time (*t*, with h as a unit). Equation (2) describes the linearized version of a pseudo-second-order model as described by Azizian [15], in which *k*_2_ is a pseudo-second-order constant and *q_e_* is the saturation concentration of the adsorbate at equilibrium:

(2) $$\frac{t}{q}=\frac{1}{k_{2}q_{e}^{2}}+\frac{1}{q_{e}}t$$

The kinetic data had a strong correlation with the adjustment proposed by the pseudo-second-order model, demonstrated by the value of the *R*^2^ of the linearization greater than 0.997. The linearization also led to the estimation of the theoretical value of *q_e_* as being 0.48 mg Cd g^−1^, which is close to the experimental data from Figure 2A. The value of *k*_2_ was estimated to be 12.56 g mg^−1^ min^−1^, which is close to other reports in the literature considering adsorptions systems alike. 

**Figure 3:**
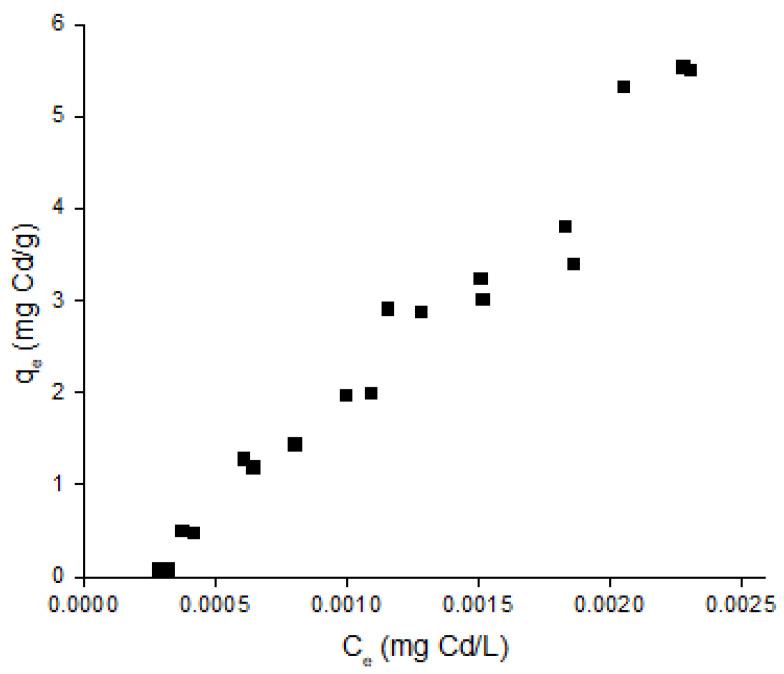
Plot of the concentration of Cd ions adsorbed onto the charcoal powder at equilibrium (*q_e_*), estimated using units of mg/g against the concentration of Cd ions in solution at equilibrium (*C_e_*) with units of mg/L, representing the isotherm curve of Cd ions onto commercial charcoal powder, considering a range of initial concentrations of Cd from 2 to 200 mg L^−1^ with assay volumes of 40.0 mL at a concentration of with 0.050 g of charcoal powder for 6 h of contact time.

The isotherm curve, prepared for an initial range of concentration of Cd ions within 2 to 200 mg L^−1^ demonstrates a typical isothermal behavior, in which an increase in the initial concentration of the adsorbate leads to higher saturation values onto the adsorbent. Given the relatively low range of concentration of the adsorbent, an initial analysis of the isotherm curve does not provide enough information for the prediction of the maximum equilibrium capacity of the adsorbent with Cd ions as adsorbates. Figure 3 highlights such observations.

The data described in Figure 3, which details all the experimental points obtained for the isotherm interpretation, were adjusted to the Langmuir and the Freundlich adsorption models. The Langmuir model, which relates the concentration of the adsorbate *C_e_* at its equilibrium time [15], with the saturation of the adsorbent at equilibrium *q_e_*, is presented as Equation (3). The constants *b* and *q_max_* on the Langmuir model are described as the Langmuir constant and the maximum saturation capacity of the adsorbent at its equilibrium, respectively. Equation (4) displays a linearized version of the Langmuir model. The Freundlich model, which is based on empirical observations between the adsorbent capacity and the concentration of the adsorbate taking into consideration the surface adsorption energy, relates *q_e_* with *C_e_* to the power of n^−1^ through the Freundlich constant A [15], as displayed as Equation (5). A common linearization of the Freundlich model is achieved through a logarithmic adjustment of the function, displayed as Equation (6).

(3) $$q_{e}=\frac{q_{max}bC_{e}}{1+bC_{e}}$$

(4) $$\frac{1}{q_{e}}=\frac{1}{q_{max}bC_{e}}+\frac{1}{q_{max}}$$

(5) $$q_{e}=AC_{e}^{\frac{1}{n}}$$

(6) $$\log q_{e}=\log A+\frac{1}{n}\log C_{e}$$

**Figure 4:**
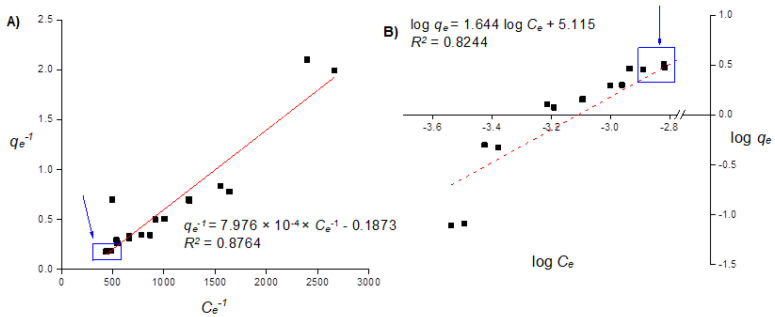
(**A**) Linearized Langmuir model, plotted with the inverse value of the Cd adsorbed capacity at equilibrium (1/*q_e_* in units of g/mg) against the inverse of the Cd ions in solution at equilibrium (1/*C_e_* in units of L/mg), and (**B**) linearized Freundlich model, plotted as the logarithm of the adsorbate capacity in terms of Cd adsorbed onto the charcoal powder (log *q_e_*) against the logarithm of the concentration of the Cd ions remaining in solution (log *C_e_*), both at equilibrium, adjusted to the isotherm profile of a range of initial concentrations of Cd from 2 to 200 mg L^−1^ with assay volumes of 40.0 mL at a concentration of with 0.050 g of charcoal powder for 6 h of contact time.

Figure 4 illustrates the correlation of the adsorption isotherm data to the linearized Langmuir and Freundlich models. If the whole data range was to be adjusted to the linearized Langmuir model, the y-axis intercept, corresponding to the value of *q_max_*, would be negative, which is impossible from the standpoint of the interpretation of the physical behavior of the phenomena involved in the process. Azizian [15] describes that this fact can be attributed to the low concentration of the adsorbent within the range taken for the analysis. Therefore, if the numerical analysis takes into consideration the values considering the initial concentration of Cd equal to or greater than 175 mg L^−1^ within the whole range studied, the regression indicates a *q_max_* value of 8.06 mg Cd g^−1^ with R^2^ = 0.8764. Similar behavior is attributed to the linearized Freundlich isotherm, in a sense that the analysis of all the data points leads to a conclusion that the adsorption phenomenon is not thermodynamically favorable due to the parameter n^−1^ being greater than 1. Nonetheless, the analysis from the data considering the initial concentration of Cd equal to or greater than 175 mg L^−1^ leads to a value of n^−1^ of 0.3266 with R^2^ = 0.8764, which fits within the range of thermodynamically favorable adsorption kinetic processes. Figure 4 highlights the whole data range as the black squares and the data points taken for the estimation of the aforementioned parameters.

**Figure 5:**
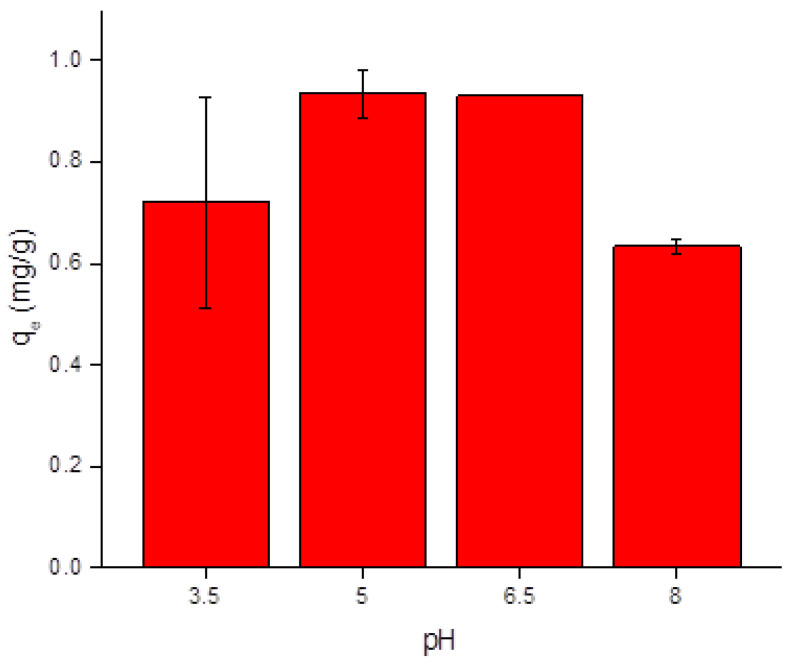
Plot of adsorbed cadmium at equilibrium (*q_e_*, measured in mg/g) against the initial pH of the aqueous solution containing Cd ions, demonstrating the effect of pH on the adsorption of Cd ions onto the commercial charcoal powder.

The effects of the medium pH were evaluated, taking into consideration a range from 3.5 to 8.0. As it can be seen from Figure 5, the confidence intervals of *q_e_* obtained from data points at pH 3.5, 5.0, and 6.5 do not allow the interpretation that they are different from a statistical standpoint. While there were some experimental discrepancies considering the error of the measurements, expressed as the error bars in Figure 5, there is an indication that the results under pH of 5 and 6.5 tend to be higher than those under alkaline conditions. The value of *q_e_* measured for an alkaline pH leads to the observation that there should be some kinetic or mass transfer difficulties in the adsorption process. According to Oyetade et al. [16], Cd ions, which behave as Cd^2+^ in pH values lower than 7.0, may be present as Cd(OH)^+^ in basic solutions. The change in the form of Cd ions may be one of the reasons that explain the difference in the adsorption behavior in different behavior, which can be summed with the potential changes in the surface characteristics of the charcoal powder.

### 3.2. Application of Charcoal and Lime Amendments: Effects on Cd Mobility

The analysis of the average composition of the *T. cacao* seedlings used in this work indicated a concentration of 0.22 mg Cd kg^−1^; meanwhile, the soil used for transplanting the seedlings contained no detectable total Cd at the beginning of the experiment. The soil, which was then dosed with Cd to a concentration of 50 mg Cd kg^−1^ also did not yield detectable losses in irrigation leachates. Therefore, the assumptions taken into consideration were that the Cd ions would adhere to soil or charcoal particles, or they would be absorbed by the seedlings through the roots.

Following two months of Cd dosing onto the soil, an analysis of the average plant material per treatment was carried out regarding different plant sections, namely the roots, stem, and leaves, as well as to the residual concentration of Cd ions in the soil. The summary of the results is presented in Table 1.

The results from Table 1 indicate that both treatments with the application of lime lead to lower mobility of Cd ions toward different parts of the seedlings. In particular, the application of lime combined with the charcoal powder demonstrated to be the single condition without detectable concentrations of Cd on the plant material. These encouraging results are in accordance with others in the literature, including the recent report by Hamid et al. [7], who demonstrated that the sole application of lime on Cd-contaminated fields led to a 25% reduction of Cd content in rice plantations. The results herein also demonstrate that the sole application of lime led to detectable concentrations of Cd onto the seedlings, which can potentially be associated with a sum of physical and chemical factors, including the lower concentration of Cd-adsorbing materials in the soil. Nonetheless, since our data collection did not consider sample taking during the 60 days of the experiment but rather just the initial and final concentration of Cd, a more definite conclusion cannot be set regarding the kinetic process of Cd mobility in the soil. The effect of the pH in the soil, thus, is evidenced to be strongly correlated to the Cd mobility into the seedlings throughout the treatment, as reviewed by Hamid et al. [7].

Table 1 also demonstrates that the results obtained from the application of the charcoal powder were not as effective in controlling Cd mobility when compared to the treatments containing lime. These results can potentially be understood given the competitive nature of adsorption of the cations in soil with the charcoal material. It has been demonstrated by studies, such as the one by Haider et al. [17], that Ca, Mg, Zn, Mn, and Al ions compete with charcoal and carbonaceous structures in the presence of Cd. Given the fact that a competitive analysis was not evaluated in the controlled adsorption assays described in Section 3.1, it cannot be firmly assumed that such ions would have preferential adsorption potentials when compared to Cd onto the charcoal particles. Nonetheless, the results describe a slightly lower concentration of Cd in the roots when compared to the control, which can potentially be linked to lower Cd mobility onto the plant materials.

The application of lime has been demonstrated in several studies to be an effective approach to control Cd mobility in pea and wheat [18], rice [19], hyacinth [20], and others. Liming has been described to not only control Cd mobility through pH shifts, but also by increasing the concentration of Ca ions in the soil, therefore enhancing the competition between ions and reducing Cd uptake by plants. The application of carbonaceous materials in Cd-contaminated soils has also been demonstrated to increase growth rates in a variety of crops, as thoroughly reviewed by Hamid et al. [7]. In this sense, the results described herein align with some other reports. Furthermore, our data describe that a combination of amendments with different mechanisms, including adsorption and pH control, may lead to lower mobility of Cd onto *T. cacao* seedlings.

## Figures and Tables

**Table 1:** pH and average distribution of total Cd in different sections of the seedlings and on the soil at the end of the data collection. ND indicates that Cd ions were not detected at a detection level of 0.5 mg Cd kg^−1^.

Treatment	Leaves (mg Cd kg^−1^)	Stem (mg Cd kg^−1^)	Roots (mg Cd kg^−1^)	Soil (mg Cd kg^−1^)	pH
Control	28.8 ± 4.0	47.4 ± 10.3	130.5 ± 18.6	24.6 ± 4.1	6.28 ± 0.5
Lime	ND	ND	13.0 ± 10.9	28.7 ± 2.9	7.46 ± 0.4
Charcoal	20.4 ± 3.7	46.6 ± 6.3	108.0 ± 14.5	18.1 ± 0.6	6.04 ± 0.7
Charcoal + Lime	ND	ND	ND	35.9 ± 1.7	7.48 ± 0.6

ND: not detected.
